# HiNO: An Approach for Inferring Hierarchical Organization from Regulatory Networks

**DOI:** 10.1371/journal.pone.0013698

**Published:** 2010-11-04

**Authors:** Mara L. Hartsperger, Robert Strache, Volker Stümpflen

**Affiliations:** Institute of Bioinformatics and Systems Biology (MIPS), Helmholtz Zentrum München – German Research Center for Environmental Health, Neuherberg, Germany; University of Birmingham, United Kingdom

## Abstract

**Background:**

Gene expression as governed by the interplay of the components of regulatory networks is indeed one of the most complex fundamental processes in biological systems. Although several methods have been published to unravel the hierarchical structure of regulatory networks, weaknesses such as the incorrect or inconsistent assignment of elements to their hierarchical levels, the incapability to cope with cyclic dependencies within the networks or the need for a manual curation to retrieve non-overlapping levels remain unsolved.

**Methodology/Results:**

We developed HiNO as a significant improvement of the so-called breadth-first-search (BFS) method. While BFS is capable of determining the overall hierarchical structures from gene regulatory networks, it especially has problems solving feed-forward type of loops leading to conflicts within the level assignments. We resolved these problems by adding a recursive correction approach consisting of two steps. First each vertex is placed on the lowest level that this vertex and its regulating vertices are assigned to (downgrade procedure). Second, vertices are assigned to the next higher level (upgrade procedure) if they have successors with the same level assignment and have themselves no regulators. We evaluated HiNO by comparing it with the BFS method by applying them to the regulatory networks from *Saccharomyces cerevisiae* and *Escherichia coli*, respectively. The comparison shows clearly how conflicts in level assignment are resolved in HiNO in order to produce correct hierarchical structures even on the local levels in an automated fashion.

**Conclusions:**

We showed that the resolution of conflicting assignments clearly improves the BFS-method. While we restricted our analysis to gene regulatory networks, our approach is suitable to deal with any directed hierarchical networks structure such as the interaction of microRNAs or the action of non-coding RNAs in general. Furthermore we provide a user-friendly web-interface for HiNO that enables the extraction of the hierarchical structure of any directed regulatory network.

**Availability:**

HiNO is freely accessible at http://mips.helmholtz-muenchen.de/hino/.

## Introduction

One of the fundamental problems in systems biology is the understanding of regulatory networks and the interdependencies between their components. Of particular importance is how transcription factors (TFs) mutually coordinate the expression of thousands of genes and noncoding RNAs (ncRNAs) [1] like microRNAs (miRNAs) or long ncRNAs in response to various stimuli. The qualitative interaction between TFs, miRNAs and their targets can be modeled in terms of directed regulatory networks.

The determination of hierarchical interdependencies within gene regulatory networks (GRNs) is important for the understanding of the potential impact of perturbations on underlying cellular processes and their correlated diseases [2], [3]. Yu and Gerstein [4] pointed out that in contrast to well investigated undirected biological networks the superior feature of GRNs is their directedness and hence their orientation towards control rather than communication. The direct implication is a hierarchical organization, where a higher-level structure results from multiple instances of lower-level structure of different types [5]. As a consequence small groups of nodes organize in a hierarchical manner to increasingly large groups on many different scales [6]–[8].

**Figure 1:**
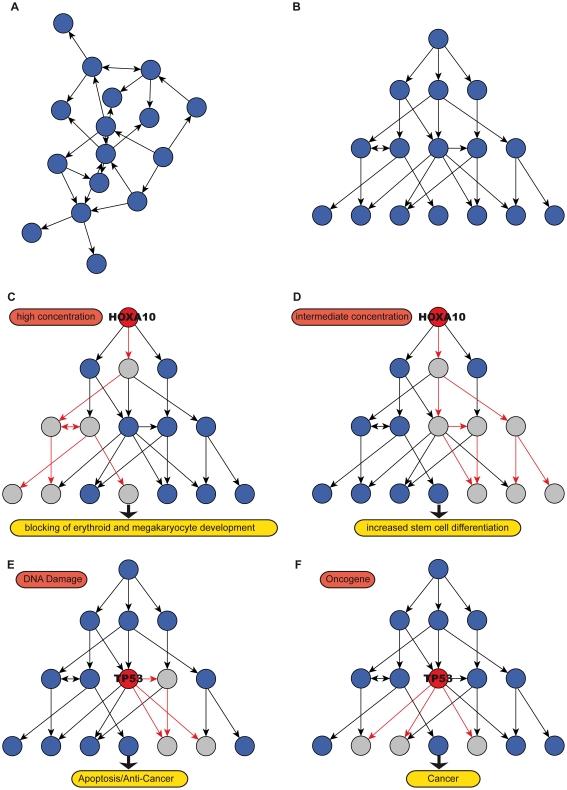
Hierarchical structure clarifies regulatory interdependencies. An abstract example of a directed regulatory network is shown (**A**) unstructured and (**B**) hierarchically structured. We schematically show in (**C**) and (**D**) that for master regulators like *HOXA10* significant downstream effects in postnatal hematopoietic development depending on different concentrations can be observed. In contrast to master regulators so called mid-level regulators can not only be mediators of different incoming regulatory signals but also influence multiple downstream components or pathways. We schematically display that *TP53* is influenced by various upstream signals such as (**E**) DNA damage or (**F**) oncogenes leading to different downstream effects such as apoptosis or development of cancer. The detailed understanding of the hierarchical topology is necessary to comprehend the dynamic behavior and the possible malfunction of regulatory networks.

It is evident that the correct determination of the overall topology of the network as well as the unambiguous prediction of the position of individual regulatory elements are essential for the comprehensive understanding of regulatory effects on a systems level (see Figure 1). For example, master regulators like *HOXA10* display significant downstream effects in postnatal hematopoietic development depending on different concentrations of the key regulator [9]. This can lead to complete blocking of erythroid and megakaryocyte development for high concentrations of *HOXA10*, whereas intermediate concentrations result in increased stem cell proliferation. In contrast to master regulators the so called mid-level regulators can not only be mediators of different incoming regulatory signals but also influence multiple downstream components or pathways. In the case of *TP53* as an important factor in cancer development it has long been known to be influenced by multiple upstream signals such as stress leading to different downstream effects such as apoptosis or development of cancer [10], [11].

The detailed understanding of the hierarchical topology is essential to understanding the dynamic behavior and the possible malfunction of regulatory networks. This can be achieved only by accurate determination of the hierarchical organization.

Several methods exist that identify the hierarchical structure within directed regulatory networks like the leaf removal algorithm [12], [13], or the breadth-first-search (BFS) method [4]. Although these methods are capable of extracting hierarchical structures from GRNs, they have several shortcomings as discussed by Jothi et al. [14]: They can either extract the hierarchical structure incorrectly or they are not scalable. Another important drawback is that they are only partly applicable to networks containing loops. However, loops are an important feature of regulatory networks [15]. Network motifs represent the building blocks of complex networks and their properties determine the local and global organization [16], [17]. Common networks motifs are feed-forward-loops (FFLs) or feedback loops (FBLs). The leaf-removal algorithm that was used for network decomposition and to infer hierarchical structures in biological networks [12], [13] is therefore not applicable to GRNs containing loops. The BFS approach presented by [4] can be applied to cyclic networks, but the inferred hierarchical structure may contain conflicts in the level assignment. Jothi et al. [14] presented a method capable of dealing with these shortcomings. However, it does not determine a clear hierarchical position for each vertex in the network rather vertices are assigned to an interval of possible positions. The algorithm does not count or enumerate all feasible topological orderings of nodes in a network because this is a NP-hard problem. It only outputs a linear ordering of nodes containing all feasible solutions rather than reporting just a single solution [14]. Subsequently the final transformation into a graph has to be done manually which is a quite cumbersome procedure for large regulatory networks.

Here, we present significant improvements of the BFS approach that directly reveal the hierarchical structure from GRNs by considering the occurrence of network motifs. This idea is implemented by expanding the BFS method with two correction steps, a “downgrade” and an “upgrade” procedure. Therefore we can determine the accurate hierarchy of a GRN that we define as its hierarchical structure without having any inconsistencies in the level assignments. To evaluate our method we applied both algorithms to the regulatory networks from *Saccharomyces cerevisiae* (YRN) and *Escherichia coli* (ERN), respectively. Our results demonstrate that the introduction of the correction steps clearly overcomes the limitations of the BFS-method. We provide a user-friendly web-interface to HiNO that enables the extraction of the hierarchical structure of any directed regulatory network.

## Materials and Methods

### Datasets

GRNs were reconstructed using publicly available data sources. We downloaded and reconstructed the GRNs for *S. cerevisiae* and *E. coli* from http://www.gersteinlab.org/proj/nethierarchy/.

The *E. coli* gene regulatory network (ERN) consisted of 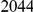 regulatory interactions between 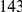 TFs and 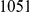 targets. The extracted TF-subgraph consisted of 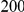 interactions between 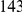 TFs with a total of 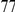 self-loops (auto-regulatory edges). The *S. cerevisiae* gene regulatory network (YRN) contained 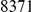 interactions involving 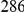 TFs and 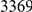 targets. The extracted TF-subgraph consisted of 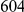 interactions involving 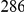 TFs with a total of 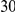 self-loops. For detailed numbers see Tables 1 and 2.

**Table 1:** Regulatory networks.

Organism	#nodes	#edges	#TFs	#genes	#targets
ERN [4]	1095	2044	143	952	1052
YRN [4]	3458	8371	286	3172	3369

Number of nodes and edges for the gene regulatory networks in E. coli (ERN) and S. cerevisiae (YRN).

**Table 2:** Transcriptional regulatory networks.

Organism	#TFs	#edges	#selfloops
TRN E. coli	143	200	77
TRN S. cerevisiae	286	604	30

Number of nodes and edges for the extracted TF-networks.

### Design and implementation

#### HiNO – an algorithm for inferring hierarchical organization from regulatory networks

**Figure 2:**
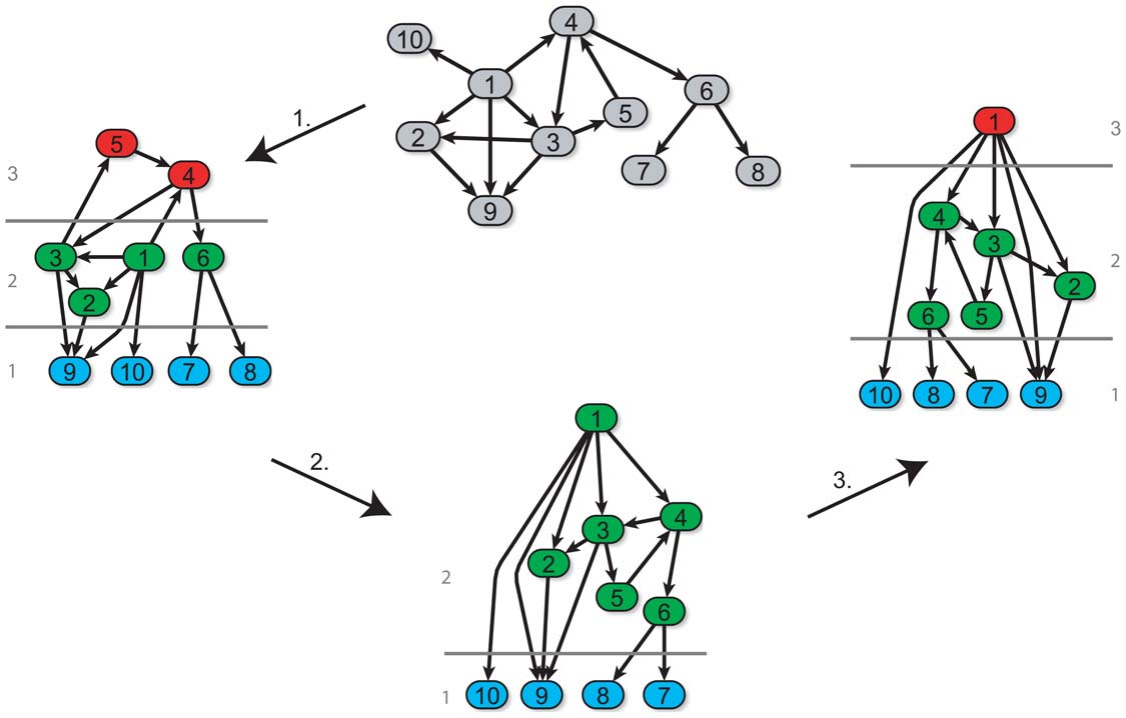
Illustration of the hierarchy extraction approach. We illustrate the approach of hierarchy extraction from directed networks. In the first step, the original network (grey) is traversed into a shortest path-tree (step1) with an initial hierarchical organization consisting of three layers. As this alone does not capture all feasible solutions we introduce two correction steps to resolve conflicts in the level assignments (e.g. node 1 is located in level 2, but it is regulating node 4 that is located in level 3). First, in the “downgrade” step each vertex is placed on the lowest level that this vertex and its regulating vertices are assigned to (step2). Thereby it is assured that each regulator has at least the same level assignment as its targets. Here, node 4 and node 5 are downgraded from level 3 to level 2 as its regulators - node 1 and node 3- are assigned to level 2. Second, vertices are “upgraded” if they have successors with the same level assignment and have no regulators themselves. Here, node 1 has no predecessor and targets on the same level (level 2). Consequently it is upgraded from level 2 to level 3 (step3). In the end HiNO determined a three layered hierarchical structure of the directed network without conflicts in the level assignment.

**Figure 3:**
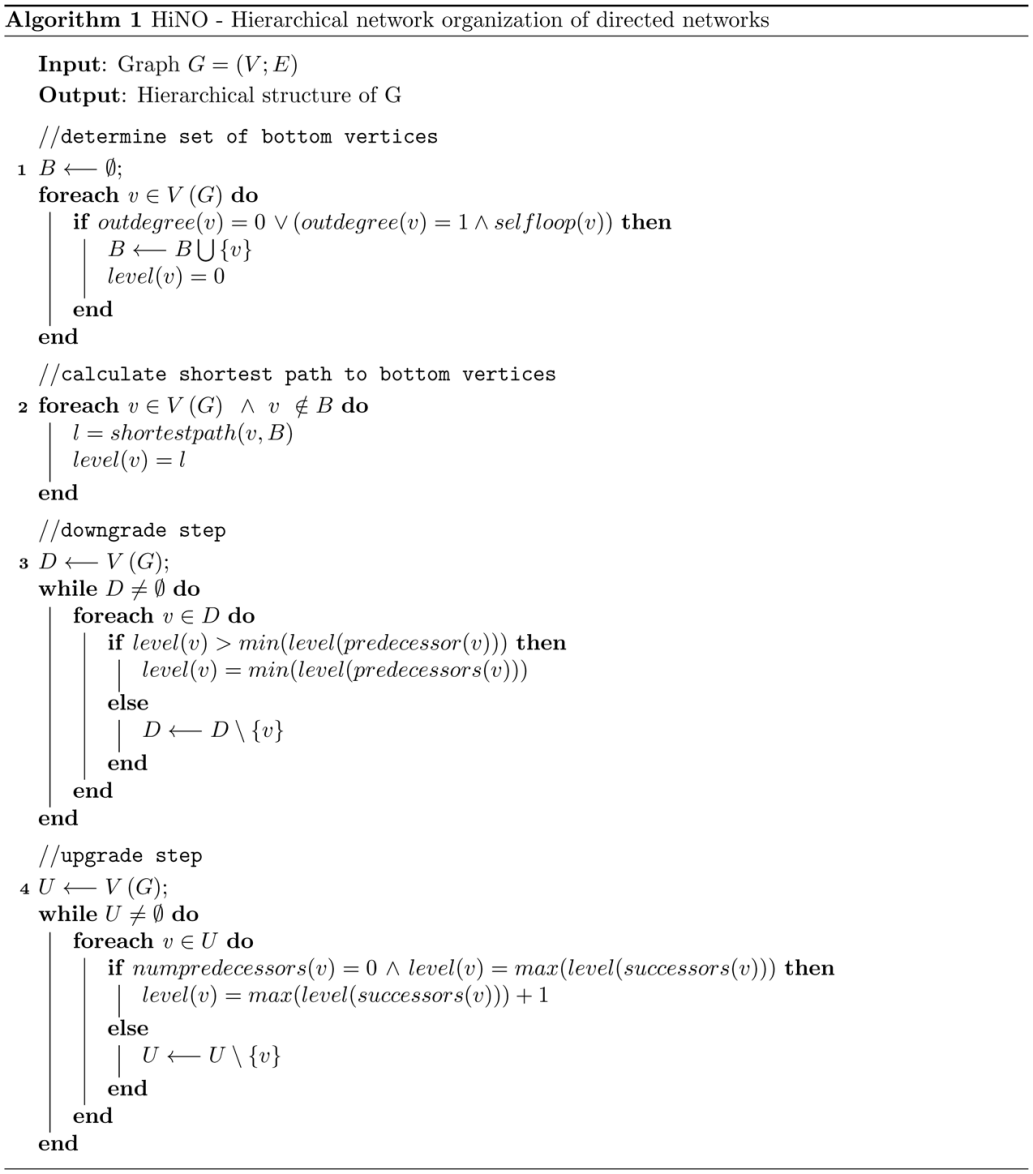
Pseudocode of the algorithm. Pseudocode for extracting the hierarchical organization of directed networks.

A directed network is a graph 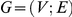 of directed edges 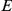 between a set of vertices 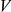. Given a directed network, our algorithm first identifies all vertices at the bottom representing the lowest level in the hierarchy. A vertex is assigned to the bottom level if and only if it does not regulate any other vertices or if it is only regulating itself (i.e. auto-regulation). In the next step, we traverse the network into a “shortest-path-tree” [18]. Here, we define the level in the hierarchy for any non-bottom vertex as its shortest distance to a bottom vertex. Now each vertex is assigned to a certain level within the hierarchy. However, in the case of occurring loops it can be observed that regulators have a lower level annotation than their targets [4]. To account for the occurrence of loops in the network (such as feed-forward loops) and to extract the underlying hierarchical structure accurately we added a recursive correction approach consisting of two steps. First, vertices are assigned to the lowest level that this and its regulating vertices are assigned to. This “downgrade” step resolves conflicts in level assignments where regulators have a lower level annotation than their targets. Second, vertices with no predecessors are placed to the next higher level if some of their successors are located on the same level. In this “upgrade” step vertices that are regulators only get the next higher level annotation than their targets. Finally, the hierarchical structure of the directed regulatory network is determined without conflicts in the vertices' level assignments. The hierarchy extraction procedure is illustrated in Figure 2. A pseudocode representation of HiNO is shown in Figure 3.

#### Web Interface

**Figure 4:**
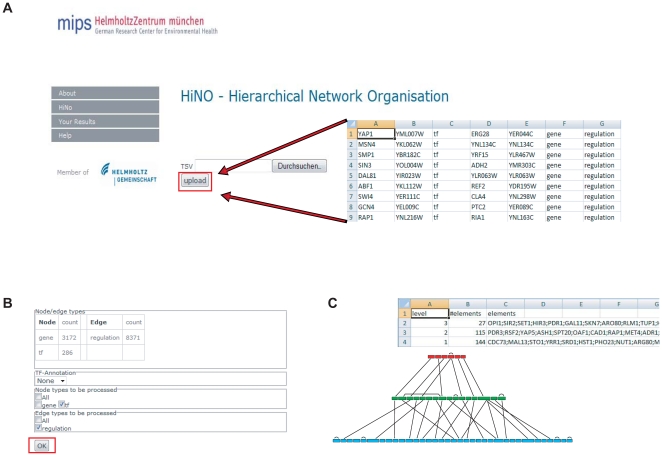
Web interface. An illustration of the user-friendly web-interface for HiNO is shown. The user can upload (**A**) a text file containing the representation of any directed network. Afterwards (**B**) the user retrieves information about node and edge types within the uploaded network and can decide which types should be used for the hierarchy extraction. Additionally, TF annotation can be added for human or mouse networks. (**C**) Information about the extracted hierarchical structure can be downloaded either as text file or in a graphical representation.

We implemented a user-friendly web application providing the functionality of HiNO, for illustration see Figure 4. Directed regulatory networks can be uploaded as text files. Users can select the node and edge types that should be used for the analysis. Additionally, TF annotation can be done for human or mouse regulatory networks using the transcription factor atlas presented by [19]. The deduced subgraph then consists of selected node and edge types only and from this its hierarchical structure is extracted. Finally, statistics on level distribution and a graphical representation of the hierarchical structure can be downloaded as text file or GraphML (an XML-based file format for graphs) file. The web interface is freely available online at http://mips.helmholtz-muenchen.de/hino/.

The source code of HiNO is available at https://sourceforge.net/projects/hino.

## Results

**Figure 5:**
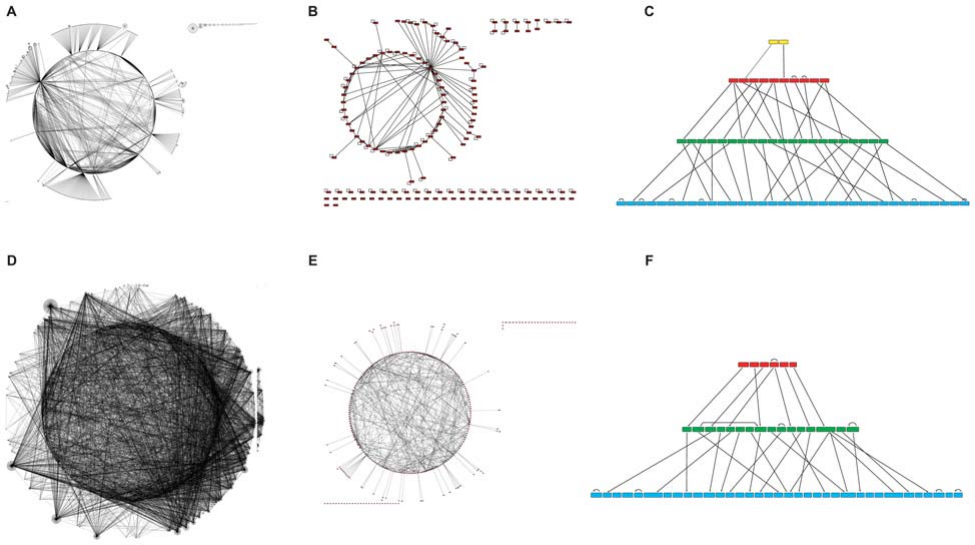
From the gene regulatory network (GRN) to the hierarchical structure. Illustration of (**A**) the GRN of E. coli with 1095 nodes and 2044 edges and (**D**) the GRN of S. cerevisiae with 3458 nodes and 8371 edges. Red nodes indicate transcription factors, green nodes genes. For extracting the hierarchical structure we deduced transcriptional regulation only. The transcriptional regulatory networks (TRNs) are shown in (**B**) for E. coli and (**E**) S. cerevisiae only. A snapshot of the hierarchical structure of the TRNs is shown for E. coli in (**C**) and for S. cerevisiae in (**F**). The TRN of E. coli has a four-layered pyramidal-shaped hierarchical structure, whereas the TRN of S. cerevisiae consists only of three layers. The different colors represent the distinct hierarchical levels: level 4– yellow; level 3– red; level 2– green; level 1– blue.

We applied HiNO to the ERN and the YRN to deduce their hierarchical structure. For this, we reconstructed the GRNs provided by [4] and subsequently extracted sub-graphs containing TFs and regulatory interactions between TFs only. The hierarchical topology is deduced only from the regulatory interdependencies of transcription factors. In Figure 5 the different analysis steps are shown, precise numbers are shown in Tables 1,2. To evaluate our method we compared the hierarchical structures extracted with the results when applying the BFS method of [4] (see Tables 3,4). The complete hierarchical structure of the ERN and the YRN can be found at http://mips.helmholtz-muenchen.de/hino/help.jsp.

**Table 3:** Level distribution of the TRN in E. coli.

Level	#elements(BFS)	#elements(HiNO)
4	4	2
3	8	10
2	42	42
1	89	89

Comparison of the level distribution of the TRN in E. coli retrieved by applying the BFS method [4] and HiNO.

**Table 4:** Level distribution of the TRN in S. cerevisiae.

Level	#elements(BFS)	#elements(HiNO)
4	8	–
3	30	27
2	104	115
1	144	144

Comparison of the level distribution of the TRN in S. cerevisiae retrieved by applying the BFS method [4] and HiNO.

### The *E. coli* regulatory network (ERN)

**Figure 6:**
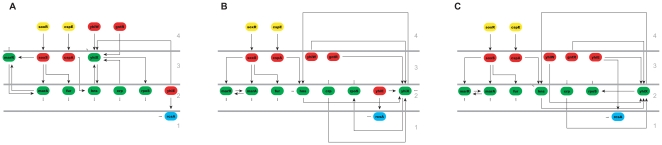
Comparison BFS-method vs. HiNO using the TRN of E. coli. A snapshot of the results (the comparison of the BFS-method and HiNO) using the TRN of E. coli is shown. Node colors indicate the level assignment in HiNO: blue - level 1; green - level 2; red - level 3; yellow - level 4. Dashed edges represent additional regulatory interdependencies (further up- and/or down-stream factors) that are not shown. The results of the BFS method are shown in (**A**). The node coloring indicates conflicts in level assignments. In (**B**) the result of the “downgrade” step of HiNO is shown. In (**C**) the result of the “upgrade” step is shown. This is also the final hierarchical assignment of the elements by HiNO.

Applying HiNO to the ERN reveals a pyramidal-shaped hierarchical structure consisting of four levels with most TFs on the bottom level (here level 1) and only a few regulators on the top (level 4). The global findings are consistent with the results when applying the method of [4] where also four levels could be identified. However, the BFS-method incorrectly assigns four TFs to the top level, whereas our method only assigns two TFs to this layer. The misassigned two elements, *gntR* and *yhiW* are placed by HiNO on the next lower level due to the correction step procedure. Both TFs regulate *yhiE* assigned to level 3 by [4]. This TF is regulated by several TFs assigned to level 2 and therefore the hierarchical structure is incorrect as a target has a higher level annotation than its regulator. In contrast, HiNO directly assigns *yhiE* to level 2 and subsequently downgrades the level annotation for its regulators *gntR* and *yhiW* to level 3. In the “upgrade” step TFs are assigned to the next higher level if they are not regulated at all and if they have targets on the same hierarchical layer. Exemplarily, this is the case for *yieH* which is upgraded from level 2 to level 3. For illustration see Figure 6, details on the level distribution are shown in Table 3.

### The *S. cerevisiae* regulatory network (YRN)

**Figure 7:**
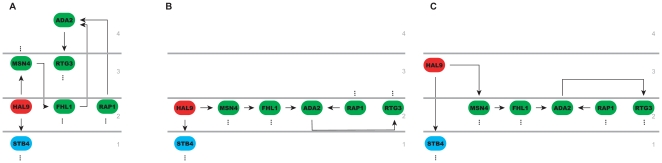
Comparison BFS-method vs. HiNO using the TRN of S. cerevisiae. A snapshot of the results (the comparison of the BFS-approach and HiNO) using the TRN of S. cerevisiae is shown. Node colors indicate the level assignment in HiNO: blue - level 1; green - level 2; red - level 3; yellow - level 4. Dashed edges represent additional regulatory interdependencies (further up- and/or down-stream factors) that are not shown. The results of the BFS method are shown in (**A**). The node coloring indicates conflicts in level assignment. In (**B**) the result of the “downgrade” step of HiNO is shown. In (**C**) the result of the “upgrade” step is shown. This is also the final hierarchical assignment of the elements by HiNO.

Applying HiNO to the YRN we extracted a pyramidal-shaped hierarchical structure consisting of three levels. In comparison to this the method of [4] reveals a four layered topology. The BFS method placed *ADA2* on the top level (level 4) although it is regulated by *RAP1* that is assigned to the second lowest level (level 2). This hierarchical assignment is inconsistent to the fact that a regulator has to be at least on the same hierarchical layer as its target. Similar conflicts can also be observed for *SPT23*, *NGG1*, *GAT1* or *MOT3* having all predecessors with lower level annotations (see Figure 7). In contrast to this HiNO assigns the correct level in the hierarchy to these TFs. In the “downgrade” step, *ADA2* is placed on the same level as its regulators *RAP1* and *FHL1* (level 2). Interdependencies are resolved subsequently by downgrading other factors such as *RTG3* or *MSN4*. Afterwards, elements are “upgraded” to the next higher level if they have no upstream elements and if they are positioned at the same layer as one of their targets. This step is illustrated in Figure 7 for *HAL9* which is upgraded from level 2 to level 3. Details on the level distribution are shown in Table 4.

### Conclusions

We presented HiNO a method that significantly improves the BFS approach presented by Yu and Gerstein [4] that directly extracts the hierarchical structure from GRNs considering the occurrence of network loops. While the BFS method returns the same hierarchical organization of the network, inconsistencies on the individual levels in local neighborhoods can be resolved by HiNO. Small, local motifs and modules define the basic dynamic properties of regulatory processes [17]. Their correct identification is necessary for the deduction of experimentally testable hypotheses deduced from large data sets. Our approach expands the BFS method with two correction steps, a “downgrade” and an “upgrade” procedure. We compared HiNO and the BFS-method using the YRN and the ERN data sets. Results demonstrate that our correction procedure clearly improves the BFS-method as it is able to reveal a hierarchical structure without inconsistencies. We provide a user-friendly web-interface that enables the extraction of the hierarchical structure of any directed network.
